# Palbociclib in combination with fulvestrant in patients with hormone receptor-positive, human epidermal growth factor receptor 2-negative advanced breast cancer: PALOMA-3 subgroup analysis of Japanese patients

**DOI:** 10.1007/s10147-018-1359-3

**Published:** 2018-11-03

**Authors:** Norikazu Masuda, Kenichi Inoue, Rikiya Nakamura, Yoshiaki Rai, Hirofumi Mukai, Shinji Ohno, Fumikata Hara, Yuko Mori, Satoshi Hashigaki, Yasuaki Muramatsu, Takashi Nagasawa, Yoshiko Umeyama, Xin Huang, Hiroji Iwata

**Affiliations:** 1 National Hospital Organization Osaka National Hospital, 2-1-14 Hoenzaka, Chuou-ku, Osaka-City, 540-0006 Japan; 2 Saitama Cancer Center, 780 Komuro, Inamachi Kitaadachi-gun, Saitama, 362-0806 Japan; 3 Chiba Cancer Center, 666-2 Nitona-Cho, Chuo-ku, Chiba, 260-8717 Japan; 4 Sagara Hospital, 3-28 Tenokuchi-cho, Kagoshima City, 892-0845 Japan; 5 National Cancer Center Hospital East, 6-5-1 Kashiwanoha, Kashiwa-shi, Chiba, 277-8577 Japan; 6 The Cancer Institute Hospital of JFCR, 3-8-31 Ariake, Koto-ku, Tokyo, 135-8550 Japan; 7 National Hospital Organization Shikoku Cancer Center, 160 Kou, Minamiumemoto-machi, Matsuyama, Ehime, 791-0280 Japan; 8 Pfizer Japan Inc, 3-22-7 Yoyogi, Shibuya-ku, Tokyo, 151-8589 Japan; 9 Pfizer Oncology, 10555 Science Center Dr, San Diego, CA 92121 USA; 10 Aichi Cancer Center Hospital, 1-1 Kanokoden, Chikusa-ku, Nagoya, 464-8681 Japan

**Keywords:** Advanced or metastatic breast cancer, Palbociclib, Fulvestrant, Japanese subgroup, Hormone receptor positive, Human epidermal growth factor receptor 2-negative

## Abstract

**Background:**

In the double-blind, phase 3 PALOMA-3 study, palbociclib–fulvestrant significantly prolonged progression-free survival versus placebo–fulvestrant in patients with hormone receptor-positive (HR+), human epidermal growth factor receptor 2-negative (HER2–) metastatic breast cancer (MBC) whose disease had progressed on prior endocrine therapy. The present study evaluated the efficacy, safety, and pharmacokinetics of palbociclib plus fulvestrant in Japanese patients enrolled in PALOMA-3.

**Methods:**

Pre/peri/postmenopausal women with HR+/HER2– MBC were randomized 2:1 to fulvestrant (500 mg) and either palbociclib (125 mg/day; 3 weeks on/1 week off; *n* = 347) or placebo (*n* = 174). Prespecified exploratory analyses compared the efficacy (data cutoff: October 23, 2015), safety, and pharmacokinetics (data cutoff: December 5, 2014) in Japanese women versus the overall population.

**Results:**

A total of 35 Japanese women were randomized to palbociclib–fulvestrant (*n* = 27) or placebo–fulvestrant (*n* = 8). Median progression-free survival was 13.6 months (95% CI, 7.5–not estimable) in the Japanese palbociclib–fulvestrant group and 11.2 months (95% CI, 5.6–not estimable) in the placebo–fulvestrant group. The most common adverse event (AE) in Japanese patients was neutropenia (all grades, 93%); no discontinuations were due to an AE. Geometric mean trough concentration values (within-subject mean steady state) for palbociclib were similar for Japanese Asian (excluding Japanese), and non-Asian patients (84.4 ng/mL, 86.3 ng/mL, and 74.8 ng/mL, respectively).

**Conclusion(s):**

The results for the overall population and Japanese patients in PALOMA-3 suggest that palbociclib plus fulvestrant was effective and well tolerated in Japanese patients with HR+/HER2‒ MBC whose disease had progressed on prior endocrine therapy (Pfizer; NCT01942135).

## Introduction

Breast cancer is the most common cancer in women worldwide and the second leading cause of cancer-related death in Asian women [1]. Although the overall mortality rate for Asian countries (including Japan) is lower compared with North America, Europe, and the Russian Federation (age-standardized mortality rate: 10.2 [Japan 9.8] vs 14.8, 16.1, and 17.2, respectively), incidence rates of breast cancer in recent generations of Asian women have increased and are approaching or surpassing the high rates historically observed in the United States, United Kingdom, and Western Europe [2].

Hormone receptor-positive (HR+)/human epidermal growth factor receptor 2-negative (HER2–) tumors are the most common form of breast malignancy. The primary first-line therapy for HR+/HER2– metastatic breast cancer (MBC) is endocrine therapy, which decreases the rate of recurrence in women diagnosed with early-stage breast cancer [3, 4]. However, many patients will eventually develop resistance to endocrine therapy, and subsequent treatment options are limited.

Targeting molecular components of the cell cycle to interrupt cell cycle progression is an effective strategy for the treatment of cancer. Cyclin-dependent kinases (CDKs) 4 and 6 promote cell cycle entry by the phosphorylation of several proteins, including retinoblastoma protein, which initiates progression from the G1 to S phase of the cycle. Palbociclib is a highly selective inhibitor of CDK4 and CDK6 that blocks progression from G1 to S phase and inhibits subsequent DNA synthesis [5, 6]. Palbociclib has been approved in the United States, the European Union, and Japan for the treatment of HR+/HER2– advanced or metastatic breast cancer in combination with endocrine therapy [7–9].

The efficacy and safety of palbociclib as first-line therapy in combination with letrozole in patients with advanced estrogen–receptor positive (ER+)/HER2– breast cancer was first demonstrated in the phase 2 PALOMA-1 study [10]. Women in the palbociclib–letrozole group demonstrated significantly longer median progression-free survival (PFS) versus those treated with letrozole alone, and adverse events (AEs) were predictable and manageable [10]. These initial findings were confirmed in the phase 3 PALOMA-2 study wherein median PFS in the palbociclib–letrozole group was 24.8 months versus 14.5 months in the placebo–letrozole group (HR, 0.58; 95% CI, 0.46–0.72; *P* < 0.001) [11]. Although the palbociclib–letrozole group had higher rates of hematologic AEs versus letrozole alone, they were successfully managed with dose reductions/interruptions, resulting in an overall favorable benefit–risk assessment.

In the phase 3 PALOMA-3 trial in patients with HR+/HER2– MBC whose disease had progressed on previous endocrine therapy, palbociclib–fulvestrant was associated with significant and consistent improvement in median PFS versus placebo–fulvestrant [12–16]. The independent data monitoring committee recommended stopping the trial early based on significant efficacy at the interim analysis (data cutoff: December 5, 2014), and updated results from the overall population have confirmed earlier findings (data cutoffs: March 16, 2015, and October 23, 2015) [7, 15].

A subgroup analysis of PALOMA-3 to assess efficacy and safety of palbociclib in premenopausal and postmenopausal Asian women (*N* = 102) showed palbociclib–fulvestrant improved PFS versus fulvestrant alone (HR, 0.485; 95% CI, 0.270–0.869; *P* = 0.0065) [17]. Palbociclib exposure was similar between Asians and non-Asians, and the safety profile of palbociclib was consistent with that previously reported [17].

Efficacy, safety, and pharmacokinetic data are available on the use of palbociclib in the Asian population; however, limited data are available on the treatment of Japanese patients with MBC. The recommended dose in Japanese patients—based on a phase 1 study of palbociclib in Japanese patients with advanced ER+/HER2– breast cancer—is 125 mg palbociclib once daily (3 weeks on/1 week off [i.e., 3/1 schedule]) in combination with 2.5 mg letrozole [18], the same as Western patients.

Although AEs reported in Japanese patients generally are consistent with the known safety profile for palbociclib, the rate of neutropenia is higher in Japanese [18, 19] and Asian patients overall than in other population groups [17]. For example, a recent analysis of the safety profile of palbociclib plus endocrine therapy in patients from the Asia Pacific (APAC) region enrolled in the PALOMA-2 and PALOMA-3 trials showed that the rate of neutropenia was consistently higher in APAC patients versus the overall population (PALOMA-2: 90.6% vs 79.5%; PALOMA-3: 94.8% vs 80.9%) [20].

We conducted prespecified exploratory analyses of the PALOMA-3 trial to evaluate the efficacy, safety, and pharmacokinetics of palbociclib–fulvestrant versus placebo–fulvestrant in premenopausal and postmenopausal Japanese patients with HR+/HER2– MBC whose disease had progressed on prior endocrine therapy. In addition, this report includes an ad hoc analysis in the overall population—including Japanese and other Asian patients—to evaluate the relationship between palbociclib exposure and body weight and to investigate risk factors associated with neutropenia, including the relationship between neutropenia and baseline neutrophil counts, palbociclib exposure, body weight, and age.

## Patients and methods

### Study design and patients

PALOMA-3—a double-blind, placebo-controlled, phase 3 clinical study conducted at 144 centers in 17 countries—evaluated the efficacy and safety of palbociclib plus fulvestrant versus placebo plus fulvestrant in premenopausal/perimenopausal or postmenopausal women (N = 521) with HR+/HER2– MBC whose disease had progressed on previous endocrine therapy. The study design has been previously published [12]. Briefly, women were eligible for the trial if they were ≥ 18 years of age, premenopausal/perimenopausal or postmenopausal, had histologically or cytologically confirmed HR+ MBC not suitable for resection or radiation therapy with curative intent, or had progressed on prior endocrine therapy (during or within 12 months after the completion of adjuvant therapy, or during or within 1 month after the end of therapy for MBC).

Patients were randomly assigned in a 2:1 ratio to receive palbociclib–fulvestrant or placebo–fulvestrant. Randomization was stratified according to the presence or absence of visceral metastasis, menopausal status at study entry, and sensitivity to prior endocrine therapy. Country or region was not a stratification factor. Palbociclib 125 mg/day or placebo was administered orally on days 1 to 21, followed by 7 days off treatment of every 28-day cycle. Fulvestrant 500 mg was administered intramuscularly on day 1 and 15 of cycle 1, and then every 28 ± 7 days thereafter starting from day 1 of cycle 1.

Premenopausal and perimenopausal women also received goserelin administered subcutaneously every 28 days during the active treatment phase. Patients continued treatment until objective disease progression, symptomatic deterioration, unacceptable toxicity, death, or withdrawal of consent, whichever occurred first.

The protocol was approved by an institutional review board/independent ethics committee, and the study was conducted in accordance with the Declaration of Helsinki. All patients provided written informed consent before the initiation of any study procedures.

### Outcomes and assessments

The primary endpoint was investigator-assessed PFS according to Response Evaluation Criteria in Solid Tumors (RECIST) v.1.1. Secondary efficacy endpoints included objective response (OR) and clinical benefit response (CBR), palbociclib plasma trough concentrations (*C*_trough_), and safety.

Tumor assessments (i.e., CT or MRI) and radionuclide bone scans were performed at baseline, every 8 weeks for the first year, then every 12 weeks after 1 year until radiographically or clinically documented progressive disease as per RECIST v.1.1, study treatment discontinuation, initiation of new anticancer therapy, or discontinuation of patient from overall study participation. Bone scans were repeated after baseline only if clinically indicated.

Trough pharmacokinetic blood samples at steady state were collected on day 15 of cycles 1 and 2 before study drug administration. Plasma samples were analyzed using a validated high-performance liquid chromatography with tandem mass spectrometry.

AE severity was graded on the basis of the National Cancer Institute Common Terminology Criteria for Adverse Events (NCI CTCAE) v.4.0.

### Statistical analyses

Prespecified exploratory analyses were performed to evaluate the efficacy, safety, and pharmacokinetics of palbociclib in Japanese patients enrolled in the PALOMA-3 study. Efficacy data are based on a data cutoff date of October 23, 2015, and study drug exposure, safety, and pharmacokinetic data on a data cutoff date of December 5, 2014.

The statistical analyses have been described previously [12]. PFS was estimated using the Kaplan–Meier method with the 95% CIs reported for both treatment arms. A log-rank test was used to compare PFS data between treatment arms. HRs were estimated from the Cox proportional hazards regression models.

OR rate and CBR rate were compared between the treatment arms using a one-sided exact test. A 95% CI for both OR rate and CBR rate was calculated.

All AEs were summarized in patients who received ≥ 1 dose of study treatment. The within-patient averages of the steady-state trough palbociclib concentrations were summarized and compared across subgroups. All analyses were performed using SAS^®^ v.9.1.3 or higher. Pearson correlation coefficient was calculated for the analysis evaluating the relationship between palbociclib *C*_trough_ and body weight as well as body surface area (BSA)/body mass index (BMI) and factors associated with post-treatment neutrophil counts (baseline neutrophil count, *C*_trough_, body weight, BSA, BMI, and age).

## Results

### Patients and study treatment

Between December 2013 and August 2014, 35 Japanese patients were enrolled and randomly assigned to palbociclib–fulvestrant (*n* = 27) or placebo–fulvestrant (*n* = 8). Demographic and baseline disease characteristics were generally similar between the Japanese and overall populations (Table 1**)**. Exceptions included Japanese patients being younger versus the overall population in the palbociclib arms (median age 53 vs 57 years) while the overall population (60%) had lower proportions of patients with an Eastern Cooperative Oncology Group performance status of 0 versus Japanese patients (100%). A greater proportion of Japanese patients versus the overall population were premenopausal/perimenopausal (48% vs 21%), and fewer Japanese patients received prior chemotherapy as metastatic treatment compared with the overall population (7% vs 31%).

**Table 1:** Patient demographics and baseline disease characteristics of the overall and Japanese population (ITT population)

Characteristic	Overall population		Japanese patients	
	PAL + FUL (*n* = 347)	PBO + FUL (*n* = 174)	PAL + FUL (*n* = 27)	PBO + FUL (*n* = 8)
Age, years				
Median (range)	57 (30‒88)	56 (29‒80)	53 (36‒77)	57 (39‒79)
< 65, *n* (%)	261 (75.2)	131 (75.3)	22 (81.5)	6 (75.0)
≥ 65, *n* (%)	86 (24.8)	43 (24.7)	5 (18.5)	2 (25.0)
Weight, median (range), kg	67.2 (35.6‒142.0)	69.8 (35.1‒126.8)	54.4 (41.0‒82.7)	52.4 (44.0‒59.0)
ECOG performance status, *n* (%)				
0	207 (59.7)	115 (66.1)	27 (100.0)	7 (87.5)
1	140 (40.3)	59 (33.9)	0	1 (12.5)
Menopausal status,^a^*n* (%)				
Pre-/peri-	72 (20.7)	36 (20.7)	13 (48.1)	4 (50.0)
Post-	275 (79.3)	138 (79.3)	14 (51.9)	4 (50.0)
Visceral metastases,^a^*n* (%)				
Yes	206 (59.4)	105 (60.3)	17 (63.0)	7 (87.5)
No	141 (40.6)	69 (39.7)	10 (37.0)	1 (12.5)
Sensitivity to prior hormonal therapy,^a,b^*n* (%)	274 (79.0)	136 (78.2)	22 (81.5)	5 (62.5)
Measurable disease, *n* (%)	268 (77.2)	138 (79.3)	21 (77.8)	8 (100.0)
Number of disease sites, *n* (%)				
1	111 (32.0)	60 (34.5)	7 (25.9)	3 (37.5)
2	99 (28.5)	50 (28.7)	12 (44.4)	1 (12.5)
3	73 (21.0)	36 (20.7)	4 (14.8)	1 (12.5)
≥ 4	62 (17.9)	26 (14.9)	4 (14.8)	3 (37.5)
Not reported	2 (0.6)	2 (1.1)	–	–
Prior chemotherapy as metastatic treatment, *n* (%)	107 (30.8)	63 (36.2)	2 (7.4)	1 (12.5)
Prior lines of therapy in the context of metastatic disease, *n* (%)				
0	84 (24.2)	45 (25.9)	7 (25.9)	3 (37.5)
1	132 (38.0)	70 (40.2)	12 (44.4)	3 (37.5)
2	90 (25.9)	43 (24.7)	5 (18.5)	2 (25.0)
≥ 3	41 (11.8)	16 (9.2)	3 (11.1)	0

*ECOG* Eastern Cooperative Oncology Group, *FUL* fulvestrant, *ITT* intent-to-treat, *PAL* palbociclib, *PBO* placebo

^a^Based on case report form data

^b^Defined as either (1) documented clinical benefit (complete response, partial response, stable disease ≥ 24 weeks) to ≥ 1 prior hormonal therapy in the metastatic setting or (2) ≥ 24 months of adjuvant hormonal therapy before recurrence

Study drug exposure analyses are presented in Table 2. Compared with the overall population, Japanese patients in the palbociclib–fulvestrant arm received a slightly lower median average daily dose of palbociclib (125 mg vs 115 mg), had a greater percentage of patients with dose reductions (32% vs 52%; most of the reductions were to the 100-mg dose), had a greater percentage of patients with a dose interruption (87% vs 100%), and had a lower median relative dose intensity (92% vs 80%).

**Table 2:** Patient exposure to palbociclib plus fulvestrant and placebo plus fulvestrant in the overall and Japanese population (as-treated population; data cutoff date: December 5, 2014)

	Overall population		Japanese patients	
	PAL + FUL (*n* = 345)	PBO + FUL (*n* = 172)	PAL + FUL (*n* = 27)	PBO + FUL (*n* = 8)
Palbociclib or placebo				
Duration of treatment,^a^ median (range), day	144 (1‒390)	120 (14‒402)	142 (42‒308)	201 (123‒301)
Average daily dose, median (range), mg	125 (81‒131)	125 (109‒129)	115 (85‒125)	125 (125‒125)
Dose reductions,^b^*n* (%)	109 (32)	3 (2)	14 (52)	0
Reduction to 100 mg	100 (29)	3 (2)	12 (44)	0
Reduction to 75 mg	27 (8)	0	4 (15)	0
Reduction to 75 mg 2/2^c^	6 (2)	0	1 (4)	0
Time to first dose reduction,^d^ median (range), day	37 (27‒240)	85 (58‒143)	34 (29‒141)	‒
Dose interruption,^e^*n* (%)	301 (87)	110 (64)	27 (100)	8 (100)
Relative dose intensity, median (range), %	92 (22‒105)	100 (69‒107)	80 (50‒100)	98 (85‒100)
Fulvestrant				
Dose interruption,^f^*n* (%)	10 (3)	2 (1)	1 (4)	0
Relative dose intensity, median (range), %	100 (50‒117)	100 (50‒108)	98 (81‒104)	100 (96‒101)

*FUL* fulvestrant, *PAL* palbociclib, *PBO* placebo

^a^Total number of days from first through last day of each study treatment

^b^Any dose reduction from the initial prescribed dose; does not include dose interruptions

^c^Palbociclib dose de-escalation to 75 mg/day 2 weeks on followed by 2 weeks off (2/2 schedule)

^d^Timed from start date of first occurrence minus first dose date of cycle 1 + 1

^e^Dose interruption defined as (1) any missing dose recorded from the case report form, (2) any gaps within 21 doses in a cycle, or (3) patient did not complete 21 doses in a cycle

^f^Dose interruption defined as (1) any missing dose recorded from case report form or (2) patient did not complete 2 doses in cycle 1

### Efficacy

**Fig. 1:**
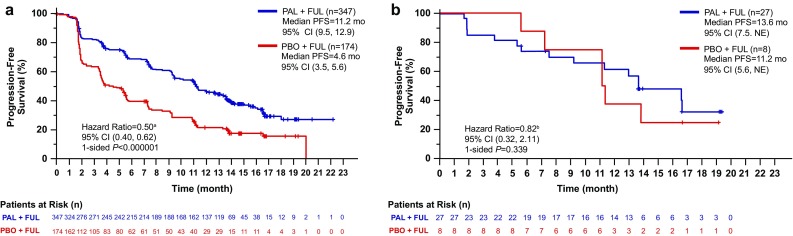
Investigator-assessed progression-free survival in patients treated with palbociclib + fulvestrant or placebo + fulvestrant in the **a** overall population and **b** Japanese patients (data cutoff date: October 23, 2015). ^a^Stratified. ^b^Unstratified. *CI* confidence interval, *FUL* fulvestrant, *NE* not estimable, *PAL* palbociclib, *PFS* progression-free survival

**Fig. 2:**
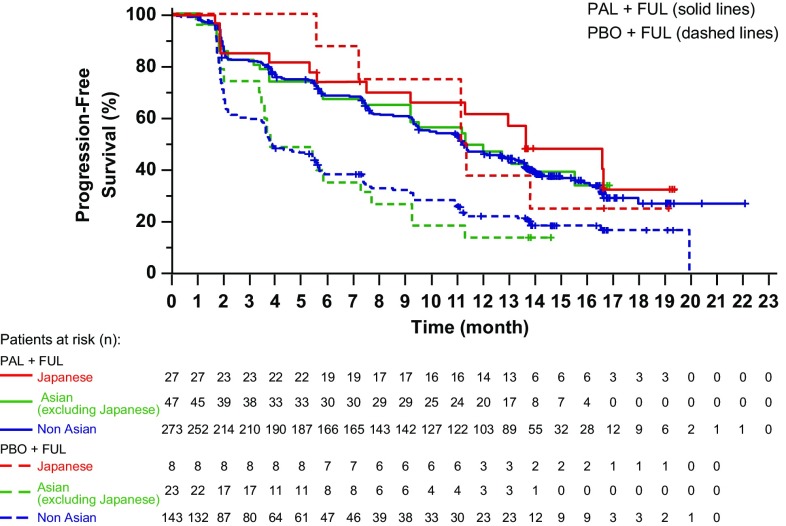
Investigator-assessed progression-free survival in non-Asian, Asian (excluding Japanese), and Japanese patients (data cutoff date: October 23, 2015). *FUL* fulvestrant, *PAL* palbociclib, *PBO* placebo

Median follow-up was 14.0 months in the palbociclib–fulvestrant arm and 14.6 months in the placebo–fulvestrant arm; 333 PFS events occurred in the overall population (200 [58%] in palbociclib–fulvestrant arm; 133 [76%] in the placebo–fulvestrant arm), and median PFS was significantly improved for palbociclib–fulvestrant (11.2 months; 95% CI, 9.5–12.9; Fig. 1a) versus placebo–fulvestrant (4.6 months; 95% CI, 3.5–5.6; HR, 0.50; *P* < 0.001). In the Japanese population, 21 PFS events occurred (15 [56%] in palbociclib–fulvestrant; 6 [75%] with placebo–fulvestrant). Median PFS for Japanese patients receiving palbociclib–fulvestrant was 13.6 months (95% CI, 7.5–NE; Fig. 1b) and 11.2 months for those receiving placebo–fulvestrant (95% CI, 5.6–NE; HR, 0.82; *P* = 0.339). PFS for Japanese patients in the palbociclib–fulvestrant arm was consistent with Asian (excluding Japanese) or non-Asian patients; however, PFS for Japanese patients in the placebo–fulvestrant arm showed a different trend compared with Asian (excluding Japanese) or non-Asian patients (Fig. 2).

For patients with measurable disease, OR rate in the overall population was higher in the palbociclib–fulvestrant versus the placebo–fulvestrant group (27%; 95% CI, 22–33 vs 11%; 95% CI, 6–17; *P* < 0.0001). In Japanese patients, OR rates were 24% (95% CI, 8–47) and 25% (95% CI, 3–65) in the palbociclib–fulvestrant and placebo–fulvestrant groups, respectively (*P* = 0.7177; Table 3). Similarly, for patients with measurable disease, CBR rate was higher in the palbociclib–fulvestrant versus placebo–fulvestrant group in the overall population (63%; 95% CI, 57–69 vs 36%; 95% CI, 28–45; *P* < 0.0001). In Japanese patients, CBR rates were 71% (95% CI, 48–89) and 88% (95% CI, 47–100) in the palbociclib–fulvestrant and placebo–fulvestrant groups, respectively (*P* = 0.9255; Table 3).

**Table 3:** Tumor response in the ITT population and in patients with measurable disease (data cutoff date: October 23, 2015)

	Overall population		Japanese patients	
	PAL + FUL	PBO + FUL	PAL + FUL	PBO + FUL
Intent-to-treat population, *n*	347	174	27	8
Best overall response, *n* (%)				
CR	0 (0)	4 (2.3)	0 (0)	0 (0)
PR	73 (21.0)	11 (6.3)	5 (18.5)	2 (25.0)
SD	181 (52.2)	72 (41.4)	17 (63.0)	6 (75.0)
≥ 24 weeks	157 (45.2)	54 (31.0)	15 (55.6)	5 (62.5)
< 24 weeks	24 (6.9)	18 (10.3)	2 (7.4)	1 (12.5)
PD	59 (17.0)	58 (33.3)	4 (14.8)	0 (0)
Indeterminate	34 (9.8)	29 (16.7)	1 (3.7)	0 (0)
OR rate (CR + PR), %	21.0	8.6	18.5	25.0
95% exact CI for OR rate^a^	16.9‒25.7	4.9‒13.8	6.3‒38.1	3.2‒65.1
Odds ratio^b^ (95% CI)	2.78 (1.56‒5.60)		0.68 (0.082‒8.96)	
One-sided *P* value^c^	0.0001		0.8204	
CBR rate (CR + PR + SD ≥ 24 weeks), %	66.3	39.7	74.1	87.5
95% exact CI for CBR rate^a^	61.0‒71.2	32.3‒47.3	53.7‒88.9	47.3‒99.7
Odds ratio^b^ (95% CI)	3.02 (2.05‒4.57)		0.41 (0.008‒4.34)	
One-sided *P* value^c^	< 0.0001		0.9057	
Patients with measurable disease, *n*	267	138	21	8
Best overall response, *n* (%)				
CR	0 (0)	4 (2.9)	0 (0)	0 (0)
PR	73 (27.3)	11 (8.0)	5 (23.8)	2 (25.0)
SD	110 (41.2)	43 (31.2)	11 (52.4)	6 (75.0)
≥ 24 weeks	95 (35.6)	35 (25.4)	10 (47.6)	5 (62.5)
< 24 weeks	15 (5.6)	8 (5.8)	1 (4.8)	1 (12.5)
PD	52 (19.5)	53 (38.4)	4 (19.0)	0 (0)
Indeterminate	32 (12.0)	27 (19.6)	1 (4.8)	0 (0)
OR rate (CR + PR), %	27.3	10.9	23.8	25.0
95% exact CI for OR rate^a^	22.1‒33.1	6.2‒17.3	8.2‒47.2	3.2‒65.1
Odds ratio^b^ (95% CI)	3.03 (1.64‒5.99)		0.94 (0.11‒12.41)	
One-sided *P* value^c^	< 0.0001		0.7177	
CBR rate (CR + PR + SD ≥ 24 weeks), %	62.9	36.2	71.4	87.5
95% exact CI for CBR rate^a^	56.8‒68.7	28.2‒44.8	47.8‒88.7	47.3‒99.7
Odds ratio^b^ (95% CI)	2.99 (1.92‒4.74)		0.36 (0.007‒4.07)	
One-sided *P* value^c^	< 0.0001		0.9255	

Stratified and unstratified odds ratio in overall population and Japanese patients, respectively

*CBR* clinical benefit response, *CI* confidence interval, *CR* complete response, *FUL* fulvestrant, *ITT* intent-to-treat, *OR* objective response, *PAL* palbociclib, *PBO* placebo, *PD* progressive disease, *PR* partial response, *SD* stable disease

^a^Exact method based on Clopper–Pearson method

^b^Odds ratio > 1 means better response in favor of palbociclib + fulvestrant

^c^One-sided exact test stratified by the presence of visceral metastases and sensitivity to prior hormonal therapy per randomization

In the intent-to-treat population, OR rate and CBR rate results were similar to those reported for patients with measurable disease. In the overall population, OR rate and CBR rate were higher with palbociclib–fulvestrant versus placebo–fulvestrant, whereas in Japanese patients the OR rate and CBR rate were lower (not statistically significant) in the palbociclib–fulvestrant versus placebo–fulvestrant arm (Table 3).

### Pharmacokinetics

**Fig. 3:**
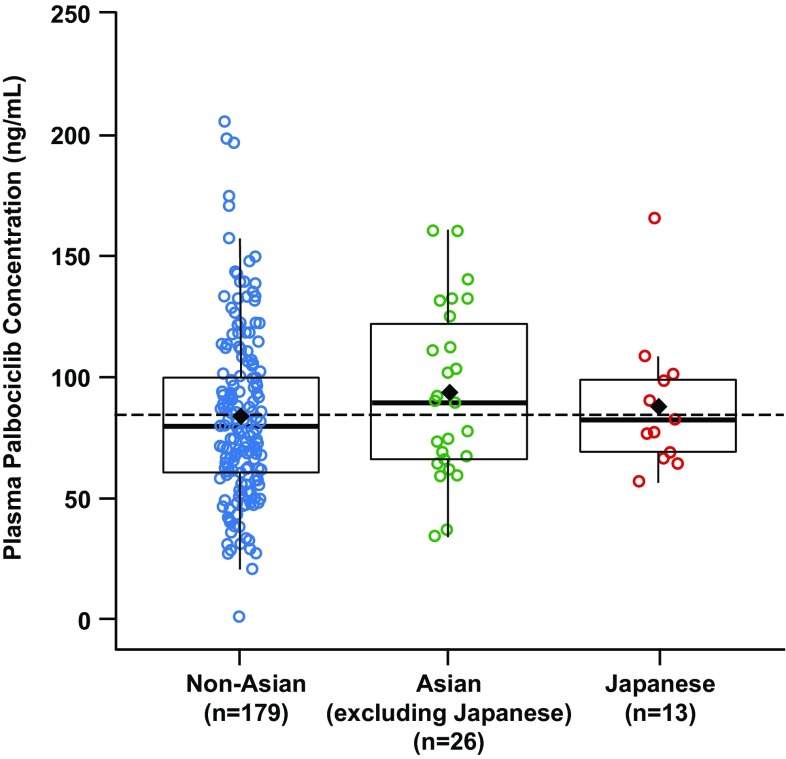
Palbociclib concentration at steady state (*C*_trough_) in non-Asian, Asian (excluding Japanese), and Japanese patients. Black diamonds represent the subpopulation arithmetic mean values and open circles represent individual patient values; the dashed black line represents the arithmetic mean value of all data from all patients; the box plot provides median and 25%/75% quartiles with whiskers to the last point within 1.5 times the interquartile range. *C*_trough_ concentration at the end of the dosing interval

**Fig. 4:**
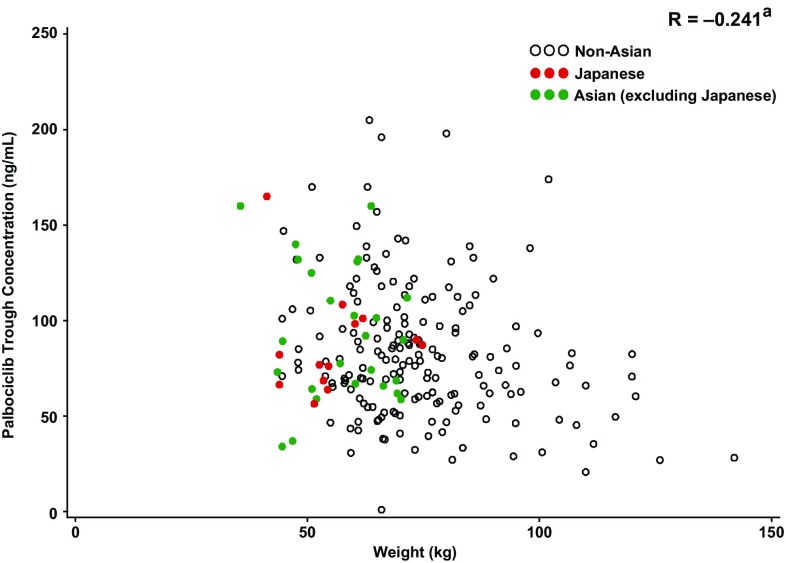
Palbociclib trough concentration at steady state (*C*_trough_) versus body weight in non-Asian, Asian (excluding Japanese), and Japanese patients. ^a^Pearson product-moment correlation coefficient. Palbociclib trough concentrations showed as within-patient *C*_trough_. *C*_trough_ concentration at the end of the dosing interval

Geometric mean steady-state palbociclib *C*_trough_ in Japanese patients was generally consistent with *C*_trough_ observed in non-Asians and Asians (excluding Japanese) [geometric mean of within-patient mean *C*_trough_ (geometric %CV): 84.4 ng/mL (28.4%) vs 74.8 ng/mL (57.2%) and 86.3 ng/mL (42.7%), respectively], indicating similar palbociclib exposure (Fig. 3). Additionally, no apparent correlation was observed between steady-state *C*_trough_ and body weight (Fig. 4) or BSA/BMI (data not shown) in Japanese, non-Asian, and Asian (excluding Japanese) patients.

### Safety

Neutropenia was the most common AE in the palbociclib arm, with higher rates reported in Japanese patients compared with the overall population (93% vs 79% of patients; Table 4). Among Japanese patients, neutropenia was typically grade ≥ 3. Similarly, Japanese patients had a higher incidence of leukopenia and thrombocytopenia (74% and 37% of patients, respectively) compared with the overall population (46% and 19%). One Japanese patient receiving palbociclib plus fulvestrant developed febrile neutropenia (grade 3) but resumed palbociclib at a lower dose after recovery. The most common nonhematologic AEs among Japanese patients in the palbociclib arm were stomatitis (44%) and rash (30%). Most nonhematologic AEs were grade 1 or 2 in severity (Table 4).

**Table 4:** AEs occurring in ≥ 15% of the overall population or in Japanese patients in either treatment arm (all causality; as-treated population: data cutoff date: December 5, 2014)

	Overall population						Japanese patients					
	PAL + FUL (*n* = 345)			PBO + FUL (*n* = 172)			PAL + FUL (*n* = 27)			PBO + FUL (*n* = 8)		
	Any grade	Grade 3	Grade 4	Any grade	Grade 3	Grade 4	Any grade	Grade 3	Grade 4	Any grade	Grade 3	Grade 4
Any AE, *n* (%)	337 (98)	202 (59)	37 (11)	153 (89)	28 (16)	3 (2)	27 (100)	18 (67)	7 (26)	8 (100)	1 (13)	0
Nonhematologic AEs, *n* (%)												
Neutropenia^a^	272 (79)	184 (53)	30 (9)	6 (4)	0	1 (< 1)	25 (93)	18 (67)	7 (26)	2 (25)	0	0
Leukopenia^a^	157 (46)	85 (25)	2 (< 1)	7 (4)	0	1 (< 1)	20 (74)	9 (33)	1 (4)	1 (13)	0	0
Thrombocytopenia^a^	67 (19)	6 (2)	2 (< 1)	0	0	0	10 (37)	0	0	0	0	0
Anemia^a^	90 (26)	9 (3)	0	17 (10)	3 (2)	0	8 (30)	1 (4)	0	2 (25)	1 (13)	0
Nonhematologic AEs, *n* (%)												
Stomatitis^a^	87 (25)	2 (< 1)	0	19 (11)	0	0	12 (44)	0	0	2 (25)	0	0
Rash^a^	48 (14)	2 (< 1)	0	8 (5)	0	0	8 (30)	1 (4)	0	0	0	0
Naso pharyngitis	25 (7)	0	0	9 (5)	0	0	7 (26)	0	0	1 (13)	0	0
Nausea	100 (29)	0	0	45 (26)	1 (< 1)	0	7 (26)	0	0	2 (25)	0	0
Fatigue	131 (38)	7 (2)	0	46 (27)	2 (1)	0	6 (22)	0	0	2 (25)	0	0
Headache	73 (21)	1 (< 1)	0	30 (17)	0	0	5 (19)	0	0	2 (25)	0	0
Pyrexia	30 (9)	1 (< 1)	0	7 (4)	0	0	5 (19)	0	0	1 (13)	0	0

*AE* adverse event, *FUL* fulvestrant, *PAL* palbociclib, *PBO* placebo

^a^Clusters of preferred terms were used to represent multiple preferred terms

More Japanese patients experienced palbociclib dose reduction due to hematologic AEs than patients in the overall population; however, no patient in the Japanese population discontinued palbociclib–fulvestrant because of AEs. AEs associated with dose reduction in Japanese patients were neutropenia (*n* = 9, 33%); neutrophil count decreased (*n* = 4, 14.8%); and anemia, aspartate aminotransferase increased, malaise, and stomatitis (all *n* = 1, 3.7%).

**Fig. 5:**
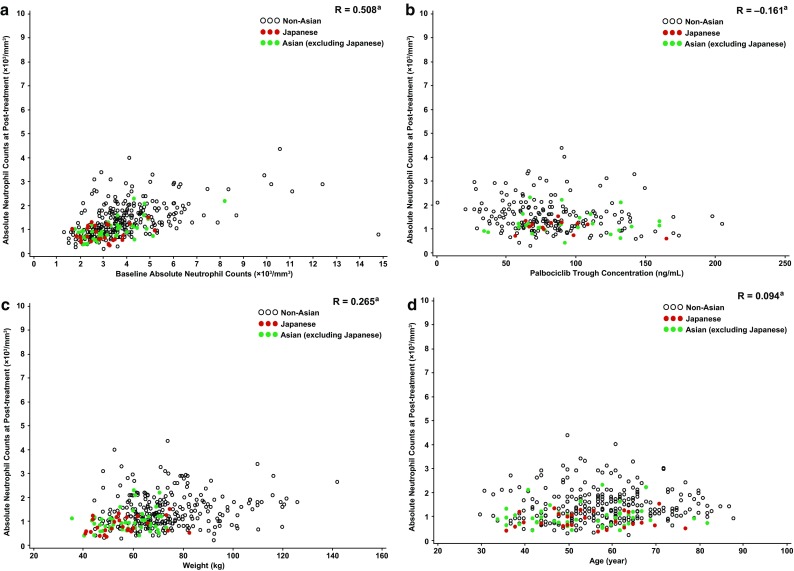
Post-treatment absolute neutrophil counts (cycle 1, day 15; data cutoff date: December 5, 2014) versus **a** baseline absolute neutrophil count, **b** palbociclib *C*_trough_, **c** body weight, and **d** age. ^a^Pearson product-moment correlation coefficient. *C*_trough_ concentration at the end of the dosing interval

The post-treatment neutrophil count (cycle 1 day 15) correlated with baseline neutrophil count in the Japanese, non-Asian, and Asian (excluding Japanese) populations (correlation coefficient = 0.508, Fig. 5a). No apparent correlation was observed in the populations for the post-treatment absolute neutrophil count versus *C*_trough_ (Fig. 5b), body weight (Fig. 5c), BSA/BMI (data not shown), or age (Fig. 5d).

## Discussion

The primary reason for this exploratory analysis was to evaluate the efficacy, safety, and pharmacokinetics of palbociclib in Japanese patients enrolled in the PALOMA-3 study and to determine whether racial differences affected the efficacy and safety of palbociclib therapy in these patients.

The addition of the first-in-class CDK 4/6 inhibitor palbociclib to fulvestrant or an aromatase inhibitor has been shown to significantly improve PFS in patients with HR+/HER2– MBC [11, 15]. A subgroup analysis of the PALOMA-3 study demonstrated that the combination of palbociclib plus fulvestrant improved PFS in premenopausal/postmenopausal Asian women with HR+/HER2– MBC whose disease had progressed on prior endocrine therapy [17]. The current exploratory analyses in Japanese patients suggest that palbociclib in combination with fulvestrant is effective in Japanese patients with HR+/HER2‒ MBC with a median PFS of 13.6 months for palbociclib–fulvestrant versus 11.2 months for placebo–fulvestrant (HR, 0.82; 95% CI, 0.32‒2.11). In addition, data showed that PFS in the palbociclib–fulvestrant arm was similar among Japanese, Asian (excluding Japanese), and non-Asian populations, indicating that all 3 populations similarly benefited from this drug combination.

Although median PFS in the placebo–fulvestrant arm in Japanese patients in PALOMA-3 was longer than previous results reported for the overall population or other populations—11.2 months (Japanese) versus 4.6 months (overall population), 5.8 months (Asian population) [17], and 3.8 months (non-Asian population) [17]—it is noteworthy that a previous phase 2 study evaluating 3 dose regimens of fulvestrant (FINDER1) reported that the median PFS was 6 months in the approved dose of fulvestrant monotherapy arm in Japanese postmenopausal women with ER+ ABC/MBC recurring/progressing on prior endocrine therapy [21].

OR and CBR rates in the placebo–fulvestrant arm were also higher in Japanese patients than in the overall population. A comparison of baseline characteristics of Japanese patients versus the overall population in the placebo–fulvestrant arm found that the rate of visceral metastases was higher (87.5% vs 60.3%) and sensitivity to prior hormonal therapy was lower (62.5% vs 78.2%) in Japanese patients than in the overall population. These data suggest that a higher percentage of Japanese patients with a poor prognosis were enrolled in the placebo–fulvestrant arm. A comparison of the baseline characteristics of Japanese patients by treatment group (placebo–fulvestrant versus palbociclib–fulvestrant) revealed a similar pattern: more patients with visceral metastases (87.5% vs 63.0%) and fewer patients with sensitivity to prior hormone therapy (62.5% vs 81.5%) were enrolled in the placebo–fulvestrant arm.

As the observed differences in baseline characteristics do not explain the longer PFS, higher OR rate, or higher CBR rate seen in Japanese patients receiving placebo–fulvestrant, it is likely that these results are related to the small number of Japanese patients (*n* = 8) in the placebo–fulvestrant treatment arm.

Palbociclib *C*_trough_ in Japanese patients was generally consistent with those in non-Asians and Asians (excluding Japanese). Palbociclib–fulvestrant was well tolerated in Japanese patients, and AEs were manageable by dose modifications and/or standard medical therapy. Neutropenia was the most commonly reported AE, with a higher rate reported in Japanese patients versus the overall population (93% vs 79%). Most nonhematologic AEs were grade 1 or 2 in severity. Similar trends were reported from Asian subpopulation analysis of PALOMA-3 [17].

Post-treatment neutrophil counts correlated with baseline neutrophil counts in Japanese, non-Asian, and Asian (excluding Japanese) populations. Japanese or other Asian populations had lower baseline neutrophil counts than non-Asian populations, which could potentially explain the higher rate of neutropenia in Japanese or other Asian patients. Data suggest that the higher incidence of neutropenia in the Japanese subgroup (vs overall population) was not related to higher palbociclib exposure (*C*_trough_), lower body weight, lower BSA/BMI, or higher age.

More Japanese patients required dose reduction in the palbociclib group resulting from hematologic AEs than patients in the overall population; however, no Japanese patient discontinued palbociclib–fulvestrant because of AEs. Importantly, dose reduction due to neutropenia did not appear to compromise PFS in the overall population in the PALOMA-3 study [22]. Additionally, although data were immature at the time of analysis, results from a recent phase 2 Japanese study also demonstrated that PFS was similar between patients requiring a dose reduction compared with patients who did not, indicating that palbociclib dose reduction did not affect PFS [19]. Finally, a recent subgroup analysis of PALOMA-2 and PALOMA-3 showed that dose intensity was lower in Asian patients compared to non-Asians due to more frequent dose reductions; however, simulated PFS profiles, based on exposure–response relationship, were similar between Asian and non-Asian patients [20]. The likely explanation for this observation is that the average concentration of palbociclib tended to be higher in Asian patients.

Although the Japanese subgroup analysis was prespecified in PALOMA-3, a limitation of our study is that the analyses lacked the power to draw definitive conclusions for some endpoints, due to the small sample size of both Japanese patient treatment arms.

These analyses of the overall and Japanese patient groups enrolled in PALOMA-3 suggest that palbociclib in combination with fulvestrant is effective and well tolerated in Japanese patients with HR+/HER2‒ MBC whose disease had progressed on prior endocrine therapy, with AEs that are manageable with dose modifications and/or standard of care medical therapies. Palbociclib should be considered as a treatment option based on results in both the overall and Japanese populations.
